# Supplementary material for: Alcohol Consumption during a Pandemic Lockdown Period and Change in Alcohol Consumption Related to Worries and Pandemic Measures
Source: Int J Environ Res Public Health. 2021 Jan 29;18(3):1220. doi: 10.3390/ijerph18031220 (PMC7908087; doi:10.3390/ijerph18031220)
Supplement: Supplementary file 1 [file ijerph-18-01220-s001.pdf]

Table S1. Percentage of decreased alcohol consumption in relation to age and gender.

Age	18–29	30–39	40–49	50–59	60–69	70+	Total
Decreased alcohol consumption (both genders)	1,455 (48%)	926 (25%)	904 (21%)	899 (19%)	631 (15%)	466 (15%)	5,281 (23%)
Women	961 (50%)	504 (23%)	487 (20%)	480 (18%)	322 (15%)	224 (15%)	2,978 (23%)
Men	494 (47%)	422 (28%)	417 (23%)	419 (20%)	309 (15%)	242 (14%)	2,303 (22%)

* Population weighted estimates (age, gender, education) for percentages

Table S2. Hazardous drinking with a medium threshold (AUDIT-C score >4 for women and >5 for men) in relation to age for all in addition to women and men, separately.

Age	18–29	30–39	40–49	50–59	60–69	70+	Total
Hazardous drinking (mt) (both genders)	1,735 (52%)	1,364 (33%)	1,554 (33%)	1,693 (32%)	1,376 (31%)	646 (18%)	8,368 (33%)
Women	1,146 (53%)	819 (33%)	923 (34%)	932 (31%)	667 (29%)	308 (18%)	4,795 (54%)
Men	589 (50%)	545 (33%)	631 (32%)	761 (33%)	709 (32%)	338 (18%)	3,573 (32%)

mt: medium threshold defined with an AUDIT-C score >4 for women and >5 for men.

Table S3. Hazardous drinking with a higher threshold (AUDIT-C score >5 for women and >6 for men) in relation to age for all in addition to women and men, separately.

Age	18–29	30–39	40–49	50–59	60–69	70+	Total
Hazardous drinking (ht)	1,119	716	781	912	719	281	4,528
(both genders)	(34%)	(17%)	(17%)	(17%)	(16%)	(8%)	(18%)
Women	696	376	395	453	304	116	2,340
	(32%)	(15%)	(14%)	(15%)	(13%)	(7%)	(16%)
Men	423	340	386	459	415	165	2,188
	(36%)	(21%)	(19%)	(20%)	(19%)	(9%)	(20%)

ht: high threshold defined with an AUDIT-C score >5 for women and >6 for men.

Table S4. Factors associated with decrease in alcohol consumption during COVID-19 pandemic phase assessed with logistic regression presented with odds ratios and 95% confidence intervals.

**Variables**	**Unadjusted OR (95% CI)**	**Adjusted OR (95% CI)**
18–29	6.7 (5.9–7.7)*	4.8 (4.2–5.5)*
30–39	3.2 (2.8–3.6)*	2.3 (2.0–2.7)*
40–49	2.3 (2.0–2.6)*	1.8 (1.5–2.0)*
50–59	1.7 (1.5–1.9)*	1.3 (1.2–1.5)*
60–69	1.3 (1.2–1.5)*	1.2 (1.0–1.3)*
70+	1.0	1.0
Female	1.0	1.0
Male	1.0 (0.97–1.1)	1.1 (1.1–1.2)*
Temporarily laid-off	1.7 (1.6–1.9)*	1.3 (1.1–1.4)*
Quarantine	1.4 (1.2–1.5)*	1.2 (1.1–1.4)*
Home office/studying	1.9 (1.7–2.0)*	1.4 (1.3–1.5)*
Economic worries	1.7 (1.6–1.9)*	1.3 (1.2–1.4)*
Health worries	1.3 (1.2–1.4)*	1.2 (1.1–1.3)*

Note. OR = Odds ratio, CI = confidence interval

* Significantly different from reference group ($p<0.05$)

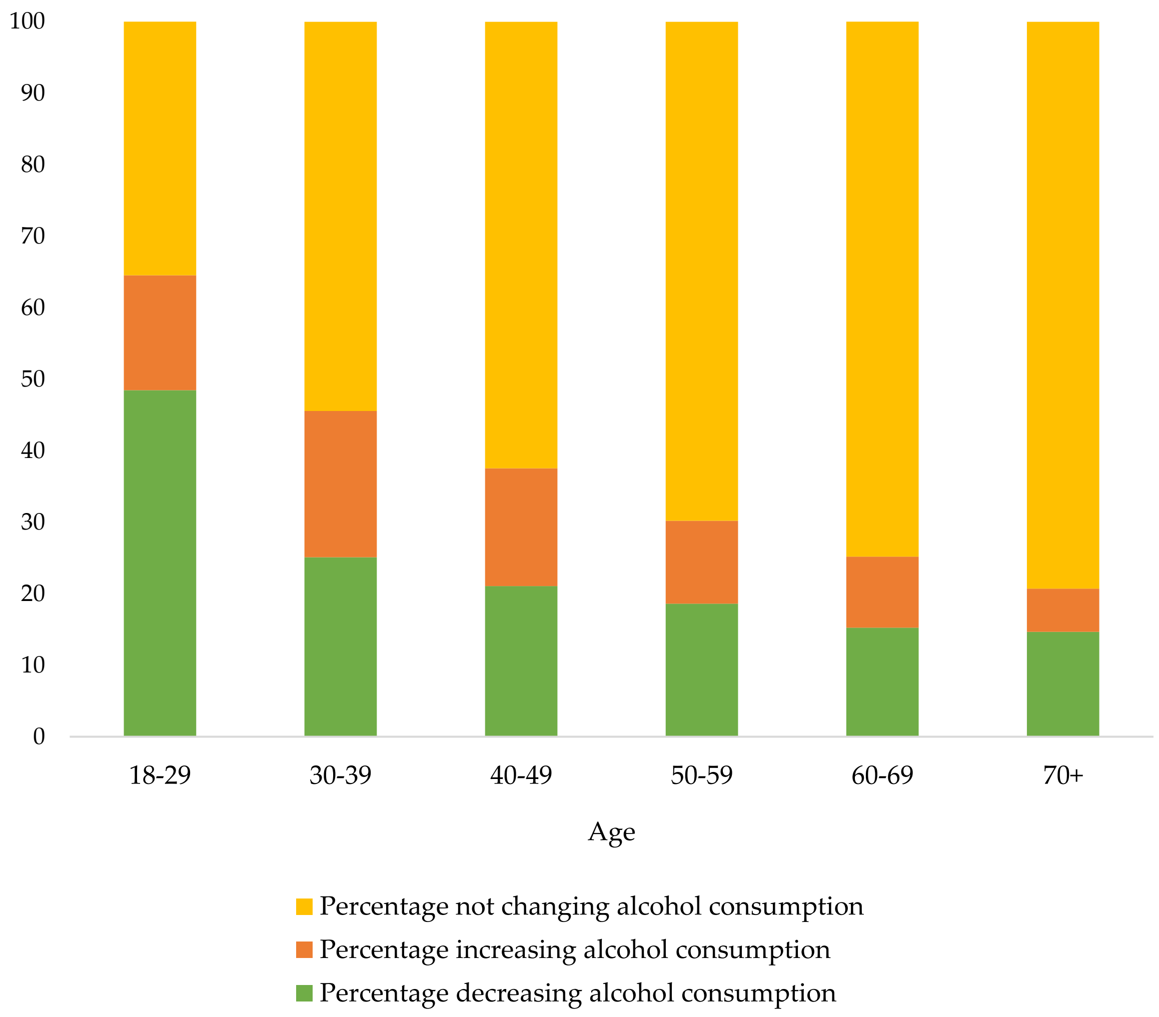

Figure S1: Percentage of decreased, increased, and unchanged alcohol consumption in relation to age.

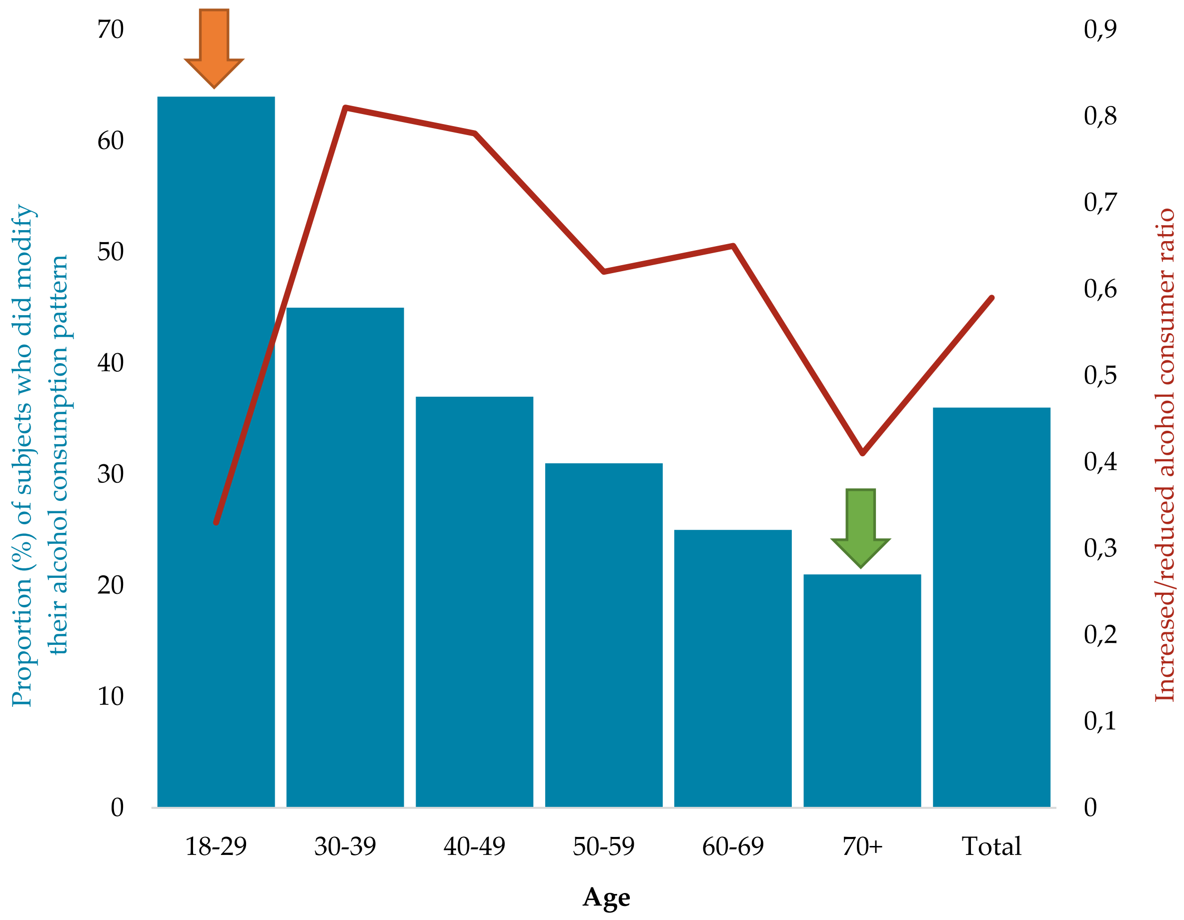

Figure S2: Proportion of subjects who did modify their alcohol consumption during the pandemic period. Blue bars/left axis indicate proportion who changed alcohol consumption while red line/right axis indicates the ratio between increased/reduced consumption. The youngest participants (orange arrow) were most likely to change alcohol consumption (most often a reduction) while the oldest participants were least likely to change consumption (green arrow), but when they did a reduction was also most common.
